# Enhancing Biomass Productivity by Forecast‐Informed Pond Operations

**DOI:** 10.1002/bit.28952

**Published:** 2025-02-07

**Authors:** Hongxiang Yan, Mark S. Wigmosta, Ning Sun, Song Gao, Michael H. Huesemann

**Affiliations:** ^1^ Energy and Environment Directorate Pacific Northwest National Laboratory Richland Washington USA; ^2^ Department of Civil and Environmental Engineering University of Washington Seattle Washington USA; ^3^ Marine Sciences Laboratory Pacific Northwest National Laboratory Sequim Washington USA

**Keywords:** biomass assessment tool, biomass forecasting, *Chlorella sorokiniana*, forecast‐informed pond operations, Huesemann algae biomass growth model

## Abstract

Microalgal cultivation for biofuels and proteins holds significant promise but faces challenges in achieving economically viable biomass productivity under variable environmental conditions. This study introduces a forecast‐informed pond operation (FIPO) system that uses numerical weather prediction (NWP) ensemble forecasts and the biomass assessment tool (BAT) to optimize daily dilution rates for enhanced biomass production. In contrast to the current practice, where fixed dilution rates are based on operator experience, the FIPO system determines the optimal dilution rate based on future weather forecasts and biomass growth conditions. Our experiments validate the effectiveness of FIPO in both short‐ and long‐term growth scenarios. In short‐term experiments, FIPO increased biomass production by 21.3% compared to batch growth and 7.4% over fixed dilution (60% every 3 days) operations. The NWP forecast‐informed operations achieved biomass production nearly identical to that using perfect weather forecasts, highlighting the accuracy of current NWP forecasts for guiding pond operations. In long‐term experiments, FIPO resulted in biomass production increases of 13.3% and 17.8% compared to two fixed dilution rates (60% every 3 days and 20% daily). These findings underscore the viability of using NWP forecasts to optimize microalgal cultivation systems. By adjusting daily dilution rates in response to forecasted weather, operators can achieve higher biomass yields and mitigate risks associated with environmental variability. This study provides a foundation for future research and practical applications in commercial‐scale microalgal production.

## 1 Introduction

Microalgal cultivation has gained significant attention for its potential to produce biofuels and proteins (Wigmosta et al. 2011; Coleman et al. 2014; Goh et al. 2019; Ganesan et al. 2020). These photosynthetic microorganisms offer numerous advantages, including rapid growth rates, the ability to grow on non‐arable land using non‐potable water, and the capacity to sequester carbon dioxide while removing waste nutrients from water. However, achieving economically viable biomass productivity remains a major challenge, particularly in outdoor cultivation systems where environmental conditions are highly variable (Davis et al. 2016, 2024). Variability in environmental conditions has increased since the 1970s due to anthropogenic climate change, with significant changes in air temperature and precipitation patterns (Thornton et al. 2014; Vasseur et al. 2014; Hou et al. 2019). For instance, Georgia experienced a heat wave in February 2023, followed by two back‐to‐back cold snaps and then additional heat waves. Similarly, the February 2021 cold snap in Texas caused cascading failures in interdependent systems, and such events are likely to become more frequent due to global warming (Cohen et al. 2021).

One critical aspect of microalgal cultivation is the operation of the cultivation system, particularly culture dilution associated with harvesting. Light attenuation in algal cultures can be severe, substantially reducing biomass productivity by limiting light penetration and encouraging respiration in the dark zones even during the day. The frequency and extent of dilution directly impact biomass concentration, light penetration, and overall productivity. Additionally, the large interface between the air and liquid culture increases the risk of biological contamination, leading to loss or failure of biomass productivity (Gao et al. 2018; Khawam et al. 2019). Current methods for managing algal cultivation systems often rely on fixed dilution rates or operational routines based on historical averages or operator experience. These approaches, while simple and easy to implement, lack the flexibility to adapt to the dynamic environmental conditions encountered in outdoor cultivation systems. Fixed dilution rates may fail to account for variations in light availability and temperature, leading to suboptimal biomass production. Moreover, these static methods do not effectively mitigate risks associated with biological contamination, which can rapidly degrade culture quality and productivity. Without the ability to dynamically adjust to changing conditions, traditional methods can result in inefficiencies, including underutilization of favorable growth periods or exacerbation of stress during unfavorable conditions. These limitations underscore the need for an adaptive and predictive system, which leverages short‐term biomass forecasting and numerical weather prediction (NWP) data to optimize cultivation strategies. By dynamically adjusting dilution rates in response to forecasted conditions, this approach can enhance productivity, reduce contamination risk, and improve the long‐term viability of microalgal cultivation systems.

To address this issue, we have developed a short‐term biomass forecasting system designed to predict biomass growth based on the weather conditions in the coming days (Yan et al. 2021, 2023), allowing operators to optimize pond operations. The forecasting system consists of the biomass assessment tool (BAT), developed at Pacific Northwest National Laboratory (PNNL) as an integrative modeling platform to evaluate potential microalgal biofuel production across the United States (Wigmosta et al. 2011; Coleman et al. 2014; Sun et al. 2020), the short‐term National Oceanic and Atmospheric Administration's (NOAA) NWP Global Ensemble Forecast System (GEFS) ensemble meteorological forecasts (Hamill et al. 2013, 2019; Bauer et al. 2015), and an ensemble data assimilation algorithm to optimally merge information from model simulation and measurements with uncertainty quantification (Yan et al. 2015, 2018; Yan and Moradkhani 2016; Abbaszadeh et al. 2018). Testing on both cold and warm strains in two different locations has shown that the biomass forecasting system can provide accurate biomass forecasts over 7 days and has promising potential to inform pond operations (Yan et al. 2021, 2023). Note that the BAT is an integrated platform designed for modeling, analysis, and data management to assess national‐scale resources and algal biomass production potential in both open‐pond and closed‐system facilities. NWP is a method of weather forecasting that employs a set of equations describing fluid flow to predict future weather based on current conditions using assimilated data.

In follow‐up research, Gao et al. (2022) adopted the biomass forecasting system and tested it operationally with field biomass growth in six bench‐scale photobioreactors replicating the weather conditions in Mesa, Arizona. Using the biomass forecasting system, they identified the optimal daily dilution rate and compared the final biomass production to that obtained under two fixed routine scenarios (batch growth and 60% harvest every 3 days). Their results suggest that using biomass forecasts, or forecast‐informed pond operation (FIPO), can improve biomass production by 20%–47% over the other two routine scenarios. In an actively growing culture using FIPO, the optimal dilution rate minimizes the dark zone and dilutes contaminants that cannot grow as fast as the algae. Consequently, long‐term continuous cultivation becomes easier, and costs associated with culture failure (e.g., inoculum preparation, cleaning, restarting) are reduced.

Despite this progress, several challenges associated with the FIPO study remain. First, the weather conditions in Gao et al. (2022) were known and controlled, equivalent to using perfect weather forecasts to drive pond operations. Therefore, the results can be considered the best achievable. In practice, NWP forecasts suffer from uncertainties and biases due to imperfect model structures, parameters, and initial conditions (Guan et al. 2022; Zhu et al. 2023). Quantifying the benefits of using FIPO in biomass production with NWP forecasts remains unclear and requires further investigation. Second, due to the cost of field studies, they could only afford a short‐term growth period (i.e., 8 days). The value of FIPO for long‐term growth (e.g., seasonal scale) still needs to be examined for commercial‐scale microalgae production.

This paper aims to address these challenges. It is the first study to investigate whether FIPO can improve biomass production using NWP forecasts over the long term. Considering the cost of real pond operations over the long term, a synthetic study is necessary to demonstrate the benefits before applying FIPO in real pond operations. This study uses a well‐validated biomass growth model within the BAT and treats its simulations as “synthetic truth” to validate the benefits of using FIPO in biomass production. Specifically, we aim to further advance the FIPO system with the use of NWP ensemble forecast data and examine biomass production on a long‐term scale to aid large‐scale biomass production.

## 2 Methods

### 2.1 BAT

The PNNL‐BAT comprises the physically based Huesemann Algae Biomass Growth Model (BGM) and the hydrodynamic Modular Aquatic Simulation System in Two Dimensions (MASS2), along with geographic information system (GIS) capabilities and data science tools. These tools include high‐resolution spatial and temporal analyses of water stress, saline water, wastewater, land, and CO_2_ resources (Supporting Information S1: Appendix A). In this proof‐of‐concept FIPO study, only the BGM and MASS2 models were used. Both models have been extensively detailed in previous studies (Perkins and Richmond 2004; Venteris et al. 2014; M. Huesemann et al. 2016; Gao et al. 2018; Yan et al. 2023). Therefore, only a brief introduction is provided here. For detailed information, readers are referred to Perkins and Richmond (2004) for MASS2 and M. Huesemann et al. (2016) for BGM.

The BGM model simulates biomass growth in algae cultures under nutrient‐replete, well‐mixed conditions, assuming constant culture pH maintained via feedback‐controlled CO_2_ addition and no growth inhibitions, such as invasive species or contamination (Supporting Information S1: Appendix B). The pond volume is divided into many equal‐volume layers perpendicular to the incident light. The BGM model uses forcing data, including light intensity (i.e., photosynthetically active radiation [PAR]) and pond water temperature. Specific microalgae growth and dark loss rates (i.e., BGM parameters) are determined from laboratory experiments as functions of culture temperature and light intensity (M. Huesemann et al. 2016). The MASS2 model is a two‐dimensional (depth‐averaged), unsteady flow and transport model that simulates hydrodynamics and water quality in ponds, rivers, and estuaries (Supporting Information S1: Appendix C). It uses a structured multi‐block grid scheme on a curvilinear grid system to discretize outdoor pond volume and employs the finite volume method to solve the energy conservation equation. The meteorological inputs for MASS2 include air temperature, dew point temperature, wind speed, air pressure, and shortwave radiation.

### 2.2 FIPO

On each growth day, the latest ensemble 1‐day ahead numerical weather forecasts are used to drive the MASS2 model, which generates ensemble 1‐day ahead pond water temperature forecasts. These temperature forecasts, along with ensemble weather data (e.g., light intensity), serve as inputs to the BGM model under various dilution rate scenarios. The system evaluates a total of 100 dilution scenarios, ranging from 0% (no operation/harvest) to 99% (nearly complete blowdown/harvest). Given 11 ensemble weather forecasts each day, the model performs 1100 simulations daily (11 weather forecasts × 100 dilution scenarios) to provide 1‐day ahead predictions. For each dilution scenario, biomass production is calculated as the ensemble mean production from the 11 weather forecasts. Operators receive 100 ensemble biomass production forecasts daily, corresponding to the 100 dilution rate options. This allows them to select the optimal dilution rate to maximize biomass production, balancing immediate harvest potential with future growth. For this study, we focus on 1‐day ahead forecasts due to their higher accuracy, assuming operators have complete flexibility to adjust dilution rates daily, from no activity to full harvesting (99%). Economic factors, such as energy and labor costs, are not included in this initial demonstration. However, advancements in NWP now enable over 95% accuracy for 3‐day ahead forecasts (Bauer et al. 2015). This FIPO system can be adapted to use 2‐ or 3‐day forecasts to optimize dilution rates over longer intervals, depending on operational needs.

**Figure 1:**
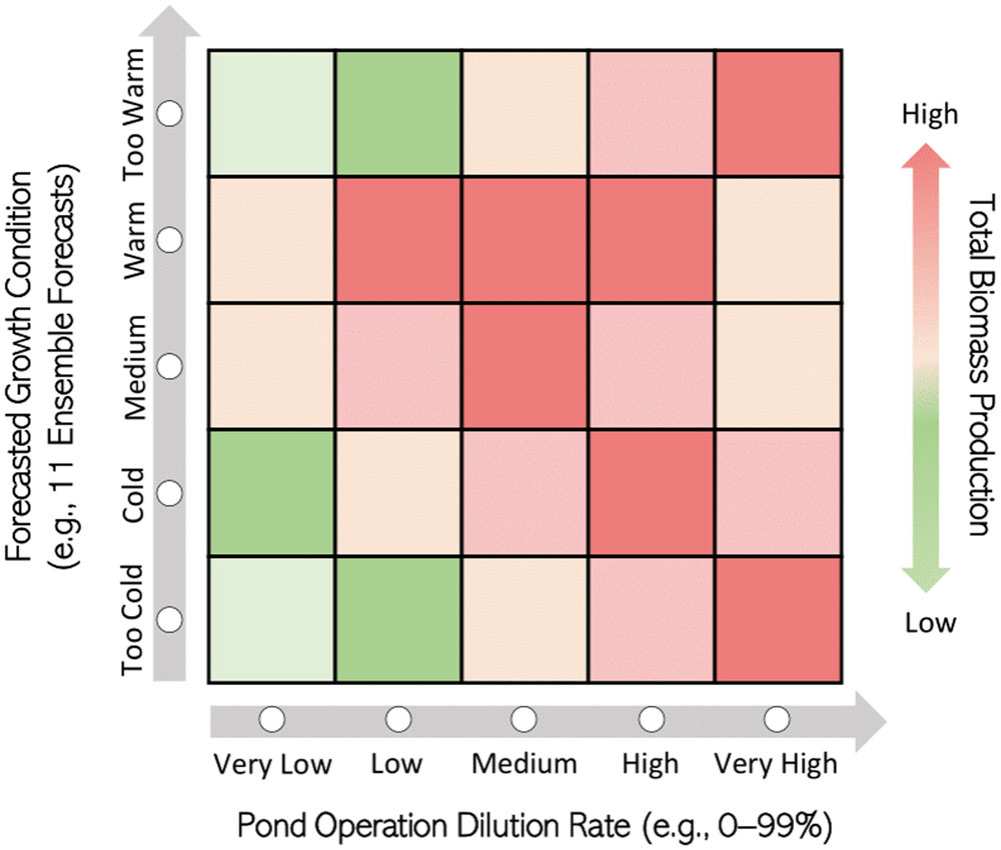
An illustration of the decision‐making matrix for forecast‐informed pond operations (FIPO).

The FIPO system is illustrated in Figure 1 as a decision‐making matrix. It shows how different pond operations (i.e., dilution rates) can lead to varying biomass production based on forecasted growth conditions (i.e., light intensity and water temperature). For example, if forecasted conditions indicate reduced biomass productivity (e.g., cold temperatures with low light intensity), a very high dilution rate is recommended to minimize biomass loss. Conversely, if conditions are ideal for exponential growth (e.g., warm water with high light intensity), a low dilution rate may be suggested if microalgae concentration does not limit light penetration, or a high dilution rate if it partially limits light penetration.

### 2.3 Microorganisms and Media

*Chlorella sorokiniana* USDOE 1412, also known as *C. sorokiniana* UTEX B 3016, was obtained from the National Alliance for Advanced Biofuels and Bioproducts (NAABB) cultivation team (Neofotis et al. 2016; Unkefer et al. 2017). From June to August 2012, *C. sorokiniana* was cultured in two indoor 800 L fiberglass raceway ponds in Mesa, Arizona, using both batch and semi‐continuous modes with two different dilution rates. Climate‐simulation ponds, which were temperature‐controlled via Labview software and illuminated with a panel of 4500 multi‐colored, computer‐dimmable LEDs to simulate outdoor light fluctuations, were used to replicate biomass growth in the outdoor ponds (M. Huesemann et al. 2017). The algae were cultured in a nutrient‐replete BG‐11 medium, maintained at pH 7 with intermittent CO_2_ sparging. Makeup water was added daily to compensate for evaporation loss and maintain the pond depth at 25 cm. The ponds operated in batch mode for 30 days from June 10 to July 10. This was followed by semi‐continuous mode with a dilution rate of 0.45/day for 13 days from July 16 to 28 and then a dilution rate of 0.21/day for 13 days from July 29 to August 10. Biomass concentrations in both ponds were manually measured once every 1–2 days using optical density at 750 nm (OD_750_) readings, which served as validation data for the BGM model. Before these pond experiments, the specific growth rate of *C. sorokiniana*, along with its dark loss rate, had been determined experimentally as a function of both temperature and light intensity in laboratory cultures (M. Huesemann et al. 2016).

### 2.4 Data Sources

For the synthetic FIPO experiments discussed in the subsequent section, historical meteorological data for Mesa, Arizona, were obtained from Phase 2 of the North American Land Data Assimilation Systems (NLDAS‐2) (Xia et al. 2015). NLDAS‐2 is a collaborative effort producing quality‐controlled, spatially and temporally consistent land surface data derived from comprehensive observations and reanalyses across the US. This meteorological data is available at daily intervals and 1/8th degree grid spacing, spanning from January 1, 1979, to the present. During the forecasting periods, numerical weather forecasts that include air temperature, wind speed, specific humidity, air pressure, and shortwave radiation were acquired from the recently released 2nd‐Generation NOAA global ensemble reforecast data set at 1° × 1° grid cell spatial resolution (Hamill et al. 2019). The once‐daily reforecast data range from 1985 to 2014 and are archived at 3‐houly intervals for lead times of 0−72 h and 6‐hourly intervals for 72−192 h. The reforecast data is statistically consistent with the currently operational National Center for Environmental Prediction (NCEP) GEFS (Hamill et al. 2013). The once‐daily GEFS reforecast was generated at 00 Coordinated Universal Time (UTC) and contained 11 ensemble members by perturbing initial atmospheric conditions (Supporting Information S1: Appendix E).

### 2.5 Two FIPO Experiments

In this study, we design two synthetic experiments to assess biomass production on both a short‐term (weekly) and long‐term (seasonal) scale. Due to the lack of long‐term biomass observations using varying daily dilution rates and the impracticality of growing biomass at all locations across the US to evaluate growth potential under all climate conditions, it is reasonable to build a “digital twin” of microalgae, known as the BGM. We use the BGM's synthetic results to assess biomass growth under different weather and operational conditions.

For the *C. sorokiniana*, the BGM has been validated against biomass measurements from both batch and semi‐continuous modes in the two growth simulation experiments mentioned in Section 2.3 (M. Huesemann et al. 2016). The field measurements of *C. sorokiniana* (DOE 1412) used in the BGM validation include OD_750_ and ash‐free dry weight (AFDW, g/L). For routine algaculture, OD_750_, despite minor operational challenges such as fouling and cleaning, is a widely used, reliable, and relatively accurate method for estimating biomass density (M. H. Huesemann et al. 2013; Edmundson and Huesemann 2015). AFDW was measured by filtering the samples onto Whatman GF/F filters, drying them at 100°C overnight, and combusting them at 520°C for 4 h. Dry weight was determined by dividing the weight of freeze‐dried algal pellets by the culture volume used to generate the pellet (Van Wagenen et al. 2012). The model performance is evaluated using the Nash−Sutcliffe efficiency (NSE) metric (Nash and Sutcliffe 1970). The NSE metric is widely used in hydrometeorological research and practice globally. It ranges from ‒∞ to 1, where a value of 1 indicates a perfect fit between observations and simulations, and a value ≤ 0 suggests that the mean of the observed values is a better predictor than the model predictions.

Compared to observations, the BGM shows very high accuracy, with NSE values of 0.99 for both ponds under batch growth mode, and 0.96 and 0.94 for the two ponds under semi‐continuous mode with different dilution rates. Supporting Information S1: Appendix D provides a detailed validation of the BGM model using measurements from both batch growth and semi‐continuous growth modes with two different dilution rates. The validation metrics demonstrate the high accuracy of the BGM in replicating observed measurements. These results confirm that the BGM is highly capable of reproducing the outdoor growth of *C. sorokiniana*. This suggests that the BGM is well‐suited for use in the FIPO studies, which will leverage NWP forecasts to further assess biomass growth under varying weather and operational conditions.

**Figure 2:**
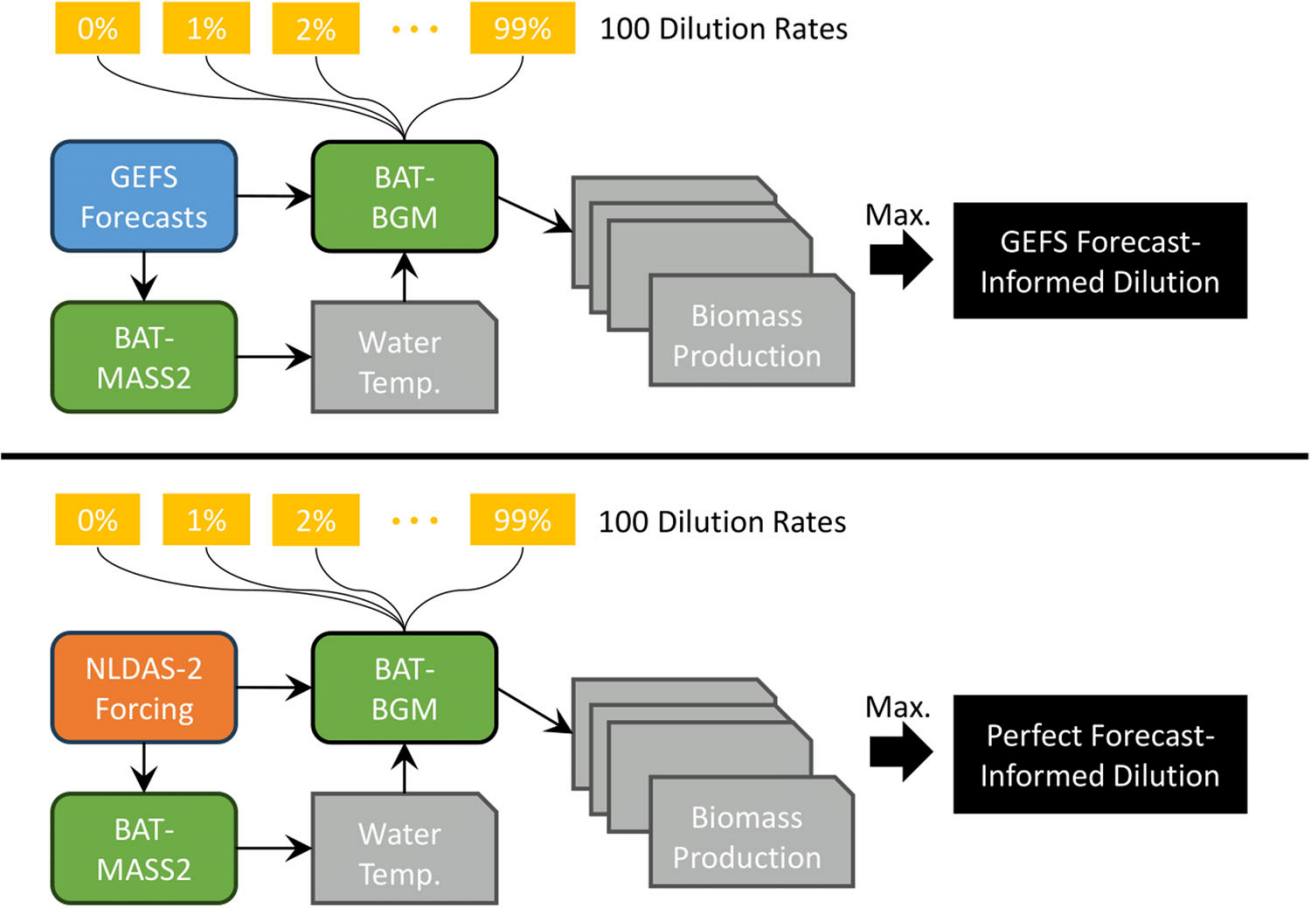
Flowchart illustrating the FIPO experiment using GEFS forecasts and perfect forecasts derived from NLDAS‐2 data.

In these FIPO experiments, the BGM simulation driven by NLDAS‐2 forcing data is considered the “synthetic truth.” The short‐term growth experiment serves as an initial step to explore the benefits of FIPO and is easier to replicate in real pond operations for research purposes. The long‐term growth experiment is intended to evaluate the implications of FIPO in industrial culture facilities, which typically operate continuously over extended periods (e.g., several months) in a semi‐continuous mode. Figure 2 illustrates the experimental setup and the integration of numerical weather forecasts with BAT. GEFS forecast data have been validated in previous studies, which identified biases in air temperature and precipitation (Guan et al. 2022; Zhu et al. 2023). We do not perform validation here because our goal is to investigate whether using state‐of‐the‐art weather forecast data, despite inherent biases, can enhance biomass production. The “perfect weather forecast” is compared to the GEFS forecast and serves as a higher benchmark to achieve. In this study, the perfect weather forecast data are derived from historical reanalysis data using NLDAS‐2 meteorological data, which integrates observations and model data through assimilation (Xia et al. 2012).

#### 2.5.1 Short‐Term Experiment

The short‐term growth experiment includes four treatments, comparing biomass productivities under different operational scenarios:

1. Treatment 1: Batch growth (baseline): The culture is run in batch mode without any operation until the harvest date, representing a large dilution at a low frequency (e.g., every 8 days). This baseline serves as the “lower bar,” where any increase in production can be attributed to the impact of dilution operations.
2. Treatment 2: Fixed dilution: The culture is diluted at a higher frequency (e.g., 60%–80% every 2–4 days), similar to the operation mode at the Arizona Center for Algae Technology and Innovation (AzCATI) for the State of the Technology (SOT) campaigns (M. H. Huesemann et al. 2024). Dilution rates can vary depending on the strain, weather, and actual growth in outdoor pond cultures. In this experiment, a dilution rate of 60% every 3 days is used to estimate biomass productivity.
3. Treatment 3: Perfect forecast‐informed dilution: The dilution rate each day is determined by the biomass forecasting system based on the next day's weather conditions. Using deterministic NLDAS‐2 data to represent perfect weather forecasts, this scenario aims to optimize the dilution rate daily, setting the “higher bar” for biomass production.
4. Treatment 4: GEFS forecast‐informed dilution: Similar to Treatment 3, the dilution rate each day is determined by the forecasting system. However, instead of deterministic NLDAS‐2 data, the real weather forecast from GEFS ensemble reforecasts is used to optimize the dilution rate. Comparing biomass production between Treatments 3 and 4 assesses whether modern weather forecasts are accurate enough to inform pond dilution.

#### 2.5.2 Long‐Term Experiment

The long‐term growth experiment also consists of four treatments. Since industrial culture facilities are operated continuously in semi‐continuous mode, no batch growth treatment is tested here.

1. Treatment 1: Fixed dilution with lower frequency: Similar to Treatment 2 in the short‐term experiment, this treatment uses a dilution rate of 60% every 3 days.
2. Treatment 2: Fixed dilution with higher frequency: Instead of diluting every 3 days, this treatment employs a daily dilution rate of 20%.
3. Treatment 3: Perfect forecast‐informed dilution: This treatment is identical to Treatment 3 in the short‐term experiment, using deterministic NLDAS‐2 data for perfect weather forecasts to optimize daily dilution rates.
4. Treatment 4: GEFS forecast‐informed dilution: This treatment mirrors Treatment 4 in the short‐term experiment, utilizing GEFS ensemble reforecasts to determine optimized daily dilution rates.

Biomass productivity under FIPO operations is compared with the two fixed dilution treatments, with any improvements in Treatments 3 and 4 attributed to the added value of biomass forecasts. Currently, pond operation decisions are typically guided by either a predefined routine or the operator's experience. In this study, we adopted fixed dilution rates of 60% every 3 days and 20% daily to reflect current microalgae cultivation practices. These dilution rates are widely used in the industry to represent typical microalgal growth conditions. Moreover, such operational approaches are commonly implemented by practitioners and have been employed in studies conducted by the AzCATI for the SOT campaigns (Gao et al. 2022; M. H. Huesemann et al. 2024). It is important to note that these specific dilution rates were chosen based on their prevalence and relevance in microalgae cultivation, rather than being the only possible configurations. Other fixed dilution rates could be explored for comparative purposes if specific operational rules or guidelines for commercial‐scale applications are identified.

## 3 Results and Discussion

### 3.1 Short‐Term Growth Comparisons

**Figure 3:**
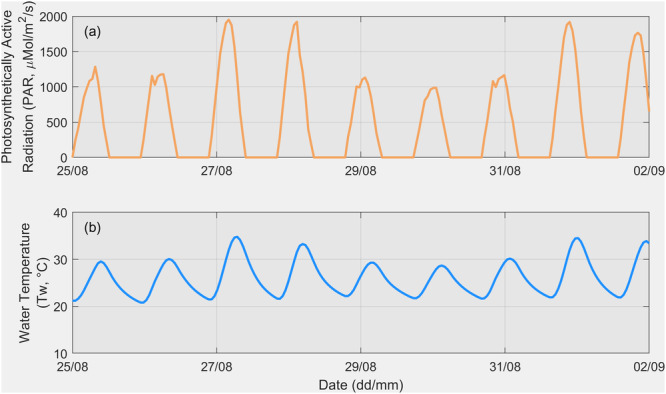
Short‐term (8.5 days) BGM model forcing data: (a) NLDAS‐2 light intensity and (b) MASS‐2 simulated outdoor pond water temperature in Mesa, Arizona.

Figure 3 presents the NLDAS‐2 PAR and simulated water temperature data, driven by NLDAS‐2 meteorological inputs, from August 25 to September 2, 2013, in Mesa, Arizona. Despite the arid conditions in this region during summer, fluctuations in PAR and pond temperature are observed over these 9 days. For example, the daily maximum PAR ranged from 987 to 1952 μMol/m²/s, with a standard deviation of 401 μMol/m²/s, while the daily maximum pond water temperature ranged from 28.7°C to 34.8°C, with a standard deviation of 2.5°C.

**Figure 4:**
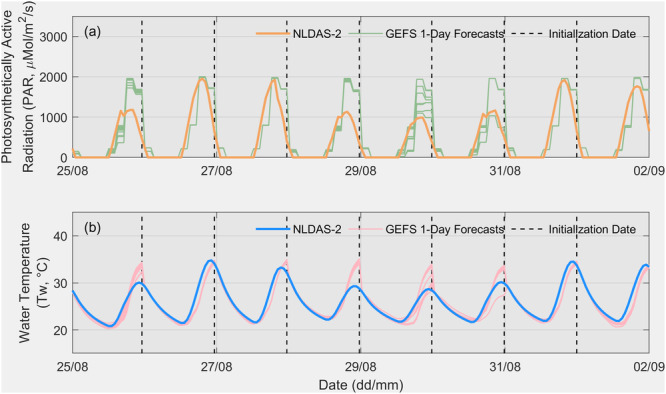
GEFS 1‐day ahead (a) light intensity ensemble forecasts and (b) MASS‐2 simulated pond water temperature ensemble forecasts driven by GEFS 1‐day ahead meteorological ensemble forecasts are presented. The plot also includes NLDAS‐2 light intensity and water temperature data driven by NLDAS‐2 forcing.

Figure 4 depicts the 11‐ensemble GEFS 1‐day ahead weather forecasts for the same period. The 3‐hourly GEFS forecasts were uniformly disaggregated into hourly time steps to run MASS2 and BGM. On average, the ensemble GEFS forecasts align well with the daily variability of PAR and pond water temperature. However, forecast accuracy varied across different days, with significant overestimation and uncertainty in the ensemble forecasts on colder days compared to warmer days during the study period. For instance, both PAR and pond water temperature forecasts showed overestimation on August 29, while the range of ensembles accurately captured the synthetic truth for August 30 and 31. Water temperature forecasts exhibited more uncertainty than PAR forecasts due to the additional meteorological data required, such as air temperature and wind speed. Limitations in the representation of orography in NWP models, including features like convection and downslope winds, further contributed to this uncertainty (Al‐Yahyai et al. 2010; Santhosh et al. 2020). Therefore, it is crucial to assess the impacts of using biased NWP forecasts on FIPO rather than relying solely on perfect weather forecasts. Notably, the bias in NWP forecasts does not necessarily translate to a similar bias in biomass simulation, as there is a nonlinear relationship between growth rate and the combined influence of pond water temperature and PAR.

**Figure 5:**
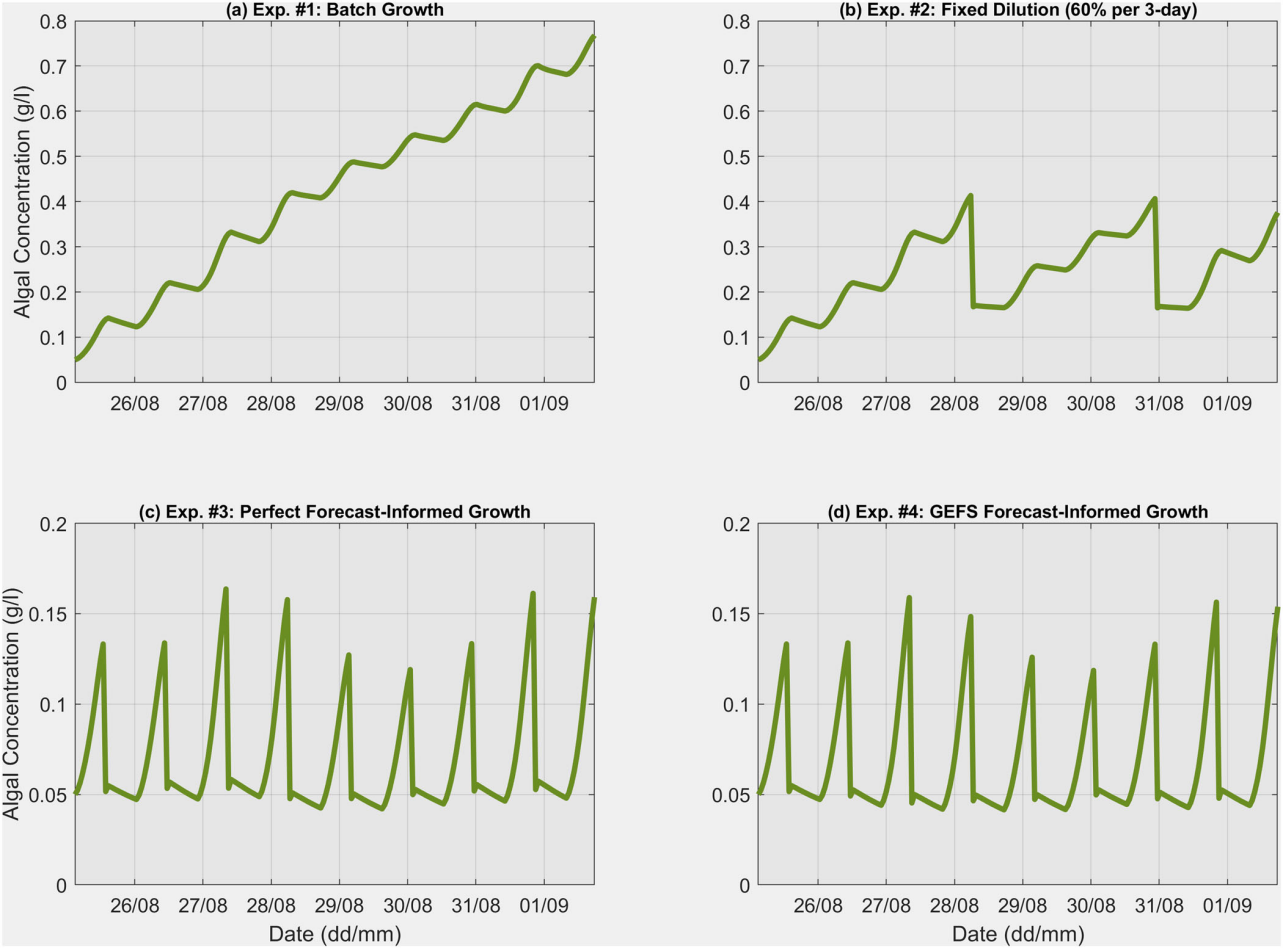
Simulated algal growth in the (a) batch growth experiment, (b) fixed dilution growth, (c) perfect forecast‐informed growth, and (d) GEFS forecast‐informed growth. In (b–d), each significant drop represents a harvest operation.

Figure 5 presents simulated biomass growth curves for the four short‐term growth experiments. In the fixed dilution growth (Figure 5b), perfect forecast‐informed growth (Figure 5c), and GEFS forecast‐informed growth (Figure 5d), each significant drop represents a harvest operation. The fixed dilution rate is 0.6 each time, while the daily optimized dilution rates for perfect forecast‐informed growth are 0.63, 0.62, 0.69, 0.71, 0.64, 0.60, 0.63, and 0.69. This variation suggests the optimal dilution rate each day to maximize biomass production. The highest dilution rate (0.71) occurs on August 28 at 18:00, in anticipation of lower PAR and pond water temperature on August 29, suggesting a larger harvest to optimize light penetration the next day. After 3 consecutive days of colder conditions, the dilution rates increase from 0.60 on August 30 to 0.63 on August 31 and 0.69 on September 1 due to warmer growth conditions, requiring a higher dilution rate to optimize biomass growth. Since this uses the perfect weather forecast, the dilution rates can be considered optimal values, with any deviations in the GEFS forecast‐informed growth attributable to biases in the weather forecasts. Closer values to these optimal rates indicate better forecast accuracy. For the GEFS forecast‐informed growth, the determined dilution rates are 0.63, 0.65, 0.73, 0.70, 0.64, 0.60, 0.66, and 0.71. Minor differences are observed, but these values are still close to those determined from the perfect forecast‐informed growth experiment, suggesting the benefits of using the imperfect GEFS forecasts.

Table 1 summarizes the total harvested biomass in the four short‐term experiments. Using batch growth, the total harvested biomass is 576 g, while the fixed dilution routine yields 651 g, a 13% increase. With the perfect forecast‐informed operation, total biomass production reaches 701 g, a 22% increase over batch growth and an 8% increase over the fixed dilution operation. These results align with our previous study (Gao et al. 2022), which showed a 20%–47% increase rate. Using the GEFS forecast‐informed operation, the total biomass production is 699 g, less than 1% below the perfect forecast‐informed operation, suggesting that current NWP forecast accuracy can achieve biomass production similar to that obtained with perfect weather forecasts. However, it is important to note that these results are from a short‐term summer period, and it is necessary to examine the outcomes in long‐term experiments, as discussed in the following section.

**Table 1:** Total harvest biomass in the four short‐term experiments.

No	Experiment description	Harvest biomass (g)	Relative diff. to num. 1
1	Batch growth	576.03	—
2	Fixed dilution (60% per 3‐day)	650.98	13.0%
3	Perfect forecast‐Informed	701.04	21.7%
4	GEFS forecast‐informed	698.90	21.3%

### 3.2 Long‐Term Growth Comparisons

**Figure 6:**
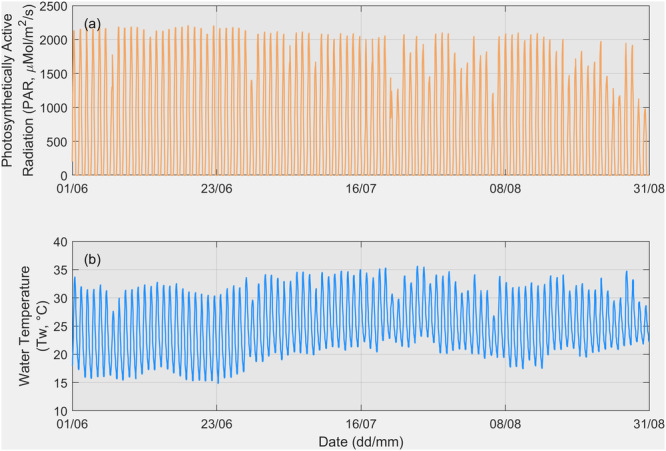
Long‐term (3‐month) BGM model forcing data includes NLDAS‐2 light intensity and MASS‐2 simulated outdoor pond water temperature (driven by NLDAS‐2 meteorological forcing data) in Mesa, Arizona.

Figure 6 illustrates the NLDAS‐2 PAR and simulated water temperature data driven by NLDAS‐2 meteorological inputs for the entire summer season from June 1 to August 31, 2013, in Mesa, Arizona. Despite the summer season, fluctuations are observed, with several days exhibiting much lower PAR and water temperatures. The daily maximum PAR values range from 987 to 2210 μMol/m²/s, with a mean of 1950 μMol/m²/s and a standard deviation of 286 μMol/m²/s. Meanwhile, the daily maximum water temperatures range from 26.9°C to 35.7°C, with a mean of 32.5°C and a standard deviation of 1.8°C. The standard deviations reflect the variability of the summer climate. This indicates that, during summer in Mesa, Arizona, the expected daily maximum PAR and pond water temperature values are approximately 1950 μMol/m²/s and 32.5°C, respectively. We have 95% confidence that the PAR values will be between 1390 and 2511 μMol/m²/s and water temperatures between 29.0°C and 35.9°C. This wide range underscores the importance of using variable dilution operations rather than a fixed dilution rate.

**Figure 7:**
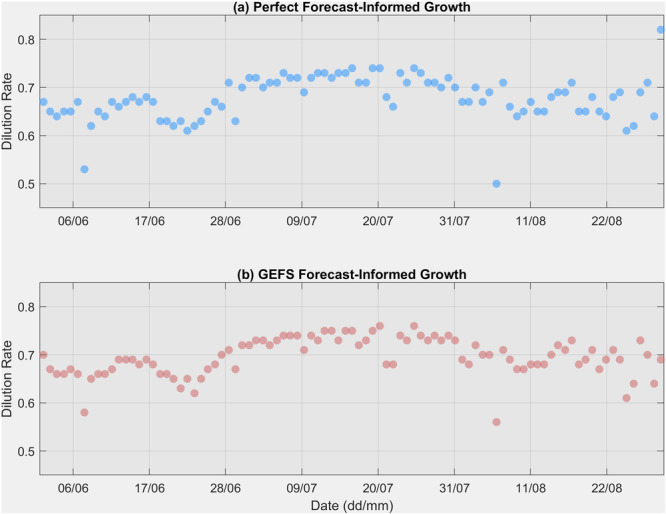
Optimized daily dilution rates based on (a) perfect forecast‐informed growth and (b) GEFS forecast‐informed growth experiments.

**Figure 8:**
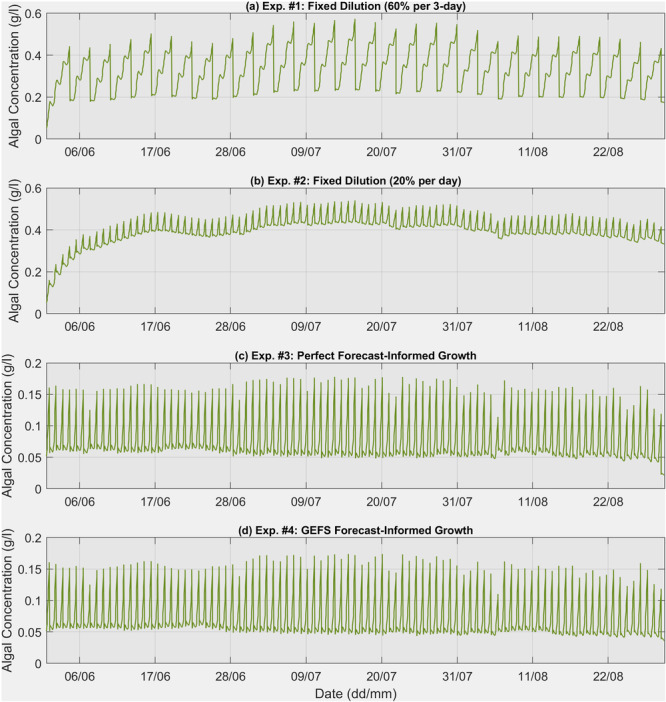
Simulated algal growth under different scenarios: (a) fixed dilution every 3 days, (b) daily fixed dilution, (c) growth informed by perfect forecasts, and (d) growth informed by GEFS forecasts. In all scenarios (a–d), each significant drop represents a harvest operation.

Figure 7 shows the optimized daily dilutions for the summer season using both perfect forecast‐informed growth and GEFS forecast‐informed growth. When using the perfect weather data (Figure 7a), the dilution rate responds to changes in weather conditions. For example, the dilution rate dropped from 0.67 on June 6 to 0.53 on June 7 as the weather was expected to warm on June 8, with the daily maximum PAR increasing from 1782 μMol/m²/s on June 7 to 2191 μMol/m²/s on June 8, and the maximum daily pond water temperature rising from 27.7°C on June 7 to 30.0°C on June 8. A similar pattern is observed on August 6, when the dilution rate dropped from 0.69 to 0.50 because the weather was expected to warm on August 7. These instances suggest a lower dilution rate when favorable weather conditions allow for higher microalgae concentrations without limiting light penetration. Conversely, the highest dilution rate of 0.82 was observed on August 30 to avoid biomass loss, as the forecast indicated continuously cooler conditions, with a daily maximum PAR of 1166 μMol/m²/s on August 31. When using GEFS forecasts (Figure 7b), a similar temporal pattern is observed, such as the drop on June 7 and August 6 and the increase on August 30, although with minor differences due to biases in the forecasts. Figure 8 displays the simulated biomass growth curves for four experiments conducted throughout the entire summer season. Each drop in the curve represents a harvest operation. In Figures 8a,b, a fixed dilution rate is used consistently throughout the summer. In contrast, Figures 8c,d show more fluctuations due to the use of varying dilution rates.

Table 2 summarizes the total harvested biomass for the entire summer season across four experiments. The two fixed dilution routines serve as the current benchmarks in practice. The total harvest biomass is 6912 g for the 0.6 fixed dilution every 3 days and 6650 g for the 0.2 fixed dilution daily. The more frequent and smaller fixed dilution rate results in about 4% less biomass compared to the less frequent and larger fixed dilution rate. However, when using the perfect forecast‐informed operation, the total biomass increases to 7866 g, representing an increase of 13.8% and 18.3% compared to the two fixed dilution rates. This represents the theoretical maximum biomass production achievable by adjusting the daily dilution rate. When using the GEFS forecast, the total biomass is 7833 g, which is less than a 1% difference compared to the perfect forecast, and still enhances biomass production by 13.3%–17.8% compared to the fixed dilution operations. These results suggest that the current NWP forecasts can be used to enhance biomass production in a long semi‐continuous growth mode.

**Table 2:** Total harvest biomass in the four long‐term experiments.

No	Experiment description	Harvest biomass (g)	Relative diff. to num. 1	Relative diff. to num. 2
1	Fixed dilution (60% per 3‐day)	6911.76	—	3.9%
2	Fixed dilution (20% per day)	6649.97	−3.8%	—
3	Perfect forecast‐informed	7866.30	13.8%	18.3%
4	GEFS forecast‐informed	7833.10	13.3%	17.8%

Beyond the lumped, deterministic comparison, we also incorporated an analysis of variance (ANOVA) to assess whether there were statistically significant differences in biomass production between the fixed dilution and FIPO experiments. This analysis was performed only for the long‐term experiment, as the short‐term experiment lacked sufficient data for ANOVA. In the 3‐month experiments, we obtained weekly biomass production data and analyzed it using ANOVA. The results indicated an *F*‐statistic value of 4.66 and a *p *< 0.05, suggesting statistically significant differences in biomass production between the fixed dilution rates and FIPO treatments.

As highlighted in the above sections, perfect forecasts represent an idealized scenario, whereas real‐time NWP forecasts, which are based on dynamic atmospheric models, can have inherent uncertainties. These uncertainties stem from model resolution, initial conditions, and physical parameterizations, which can lead to forecast errors (Guan et al. 2022; Zhu et al. 2023). The accuracy of NWP forecasts tends to decrease with forecast lead time, and small errors in temperature, solar radiation, and wind speed forecasts can have an impact on biomass productivity predictions. However, it is important to note that errors in the NWP forecast do not translate linearly into errors in biomass predictions. Additionally, the error is temporally nonstationary due to the simulation of water temperature using the MASS2 hydrodynamic model, while the biomass growth rate is influenced by both water temperature and solar radiation. While NWP forecasts are bound to contain errors, it is important to emphasize that their accuracy has been steadily improving over time (Bauer et al. 2015).

The primary aim of incorporating perfect forecasts in this study was to establish a benchmark for evaluating the performance of state‐of‐the‐art NWP forecasts, which inherently contain errors, in guiding pond dilution and enhancing biomass production. This comparison helps assess how closely the outcomes based on real‐time NWP forecasts align with those derived from perfect weather forecasts. Our results suggest that using the GEFS NWP model yields significantly better results than applying fixed dilution rates, with performance that is only slightly less optimal compared to using perfect weather forecasts. This highlights the potential value of using current NWP forecasts, despite their inherent errors, in guiding pond dilution strategies for biomass production. However, it is important to note that we used 1‐day‐ahead forecast data in this study, as it generally provides the most accurate predictions. The performance and accuracy of NWP forecasts, particularly over longer time horizons (e.g., 5 days), could vary, and we expect the results to differ depending on the forecast lead time and geographic location.

## 4 Conclusions

This study addresses the challenges of enhancing microalgal biomass productivity under variable environmental conditions by using the FIPO system. The implementation of FIPO, using NWP ensemble forecasts, demonstrated significant potential for improving biomass production over traditional fixed dilution routines.

The results of our experiments validate the effectiveness of FIPO in both short‐term and long‐term growth scenarios. In the short‐term growth comparisons, FIPO achieved a biomass production increase of 22% over batch growth and 8% over fixed dilution operations. The use of GEFS forecast‐informed operations yielded biomass production nearly identical to that achieved with perfect weather forecasts, indicating the high accuracy of current NWP forecasts for operational decision‐making. In long‐term experiments, FIPO resulted in biomass production increases of 13.8% and 18.3% compared to the two fixed dilution rates. The total biomass achieved with FIPO using perfect forecasts was 7866 g, while the GEFS forecast‐informed operation resulted in 7833 g, demonstrating the capability of NWP forecasts to closely approximate optimal conditions.

These findings underscore the viability of using NWP forecasts to inform and optimize microalgal cultivation systems. By adjusting daily dilution rates in response to forecasted weather conditions, operators can achieve higher biomass yields and reduce the risks associated with environmental variability. This study provides a foundation for future research and practical applications in commercial‐scale microalgal production, highlighting the importance of integrating advanced forecasting systems into agricultural practices for sustainable and efficient biomass production. The integration of recent advances in biomass modeling (Sun et al. 2020; Davis et al. 2024), such as the BAT, short‐ to long‐term NWP forecasts (Guan et al. 2022; Hamill et al. 2022), and data assimilation algorithms (Ahmadalipour et al. 2017; Yan et al. 2017; Zarekarizi et al. 2021), makes biomass forecasting a promising added‐value technique for optimizing the biomass production process (Yan et al. 2021, 2023; Gao et al. 2022). Short‐term biomass forecasts (daily to weekly) have the potential to effectively guide operational decisions, such as adjusting dilution rates and managing production logistics. Meanwhile, long‐term biomass forecasts (monthly to seasonal) have the potential to inform broader production strategies, including strain selection and pond water depth optimization. Future research are also needed to comprehensively understand the impact of NWP forecast errors on biomass predictions across the contiguous United States, particularly at potential cultivation sites identified by the BAT system.

Future research could also explore how forecast errors vary seasonally and how they might affect the productivity of different strains, such as cold‐tolerant versus warm‐tolerant strains, which may respond differently to forecast uncertainties. The BAT system offers flexibility to incorporate additional strains, requiring only the replacement of the parameter file for each new strain in the BGM. Concurrently, efforts are underway to expand the strain parameter database through controlled laboratory experiments to characterize growth rates under diverse weather conditions. This process will enhance the applicability of FIPO across a wider range of microalgal species. Regarding long‐term forecasting, to the best of our knowledge, the potential benefits of long‐term biomass forecasts (monthly to seasonal) in guiding cultivation strategies remain largely unexplored. We recommend future studies leverage long‐term forecast data to evaluate its utility in optimizing decisions, such as identifying which strain and pond depth combinations are likely to yield the highest productivity in the upcoming month or season.

Our study focused on optimizing biomass productivity using the FIPO system under ideal conditions, assuming no contamination and full nutrient availability. While the BGM model and FIPO framework are designed to maximize biomass production, factors such as contamination risk were not explicitly addressed, as they fall outside the scope of this initial study. That said, FIPO has the potential to reduce contamination stress by minimizing the “dark zone” within the pond. Additionally, the higher dilution rate in the FIPO system, compared to batch cultures, facilitates the gradual removal of slower‐growing contaminants, thereby reducing contamination. It is important to note that this concept is independent of harvesting operations.

Additionally, although FIPO can enhance biomass production by 10%−20% and reduce the potential for pond contamination and growth failure, its economic implications warrant careful consideration, especially for commercial‐scale applications. While FIPO's ability to significantly improve biomass productivity has the potential to lower production costs, the implementation of higher dilution rates may lead to increased energy consumption for water movement, higher labor costs, and greater demands on water resources. Even if the culture medium is recycled, these operational costs may exceed those of fixed dilution operations, potentially offsetting the economic benefits. Moreover, a comprehensive techno‐economic analysis (TEA) is essential to quantify the cost reductions and efficiency improvements associated with FIPO, particularly in large‐scale biomass production systems. This analysis should explore how FIPO can be adapted to optimize resource use and enhance the overall economic viability of microalgal cultivation. Understanding these trade‐offs will provide useful insights into the broader applicability of FIPO for commercial‐scale operations.

## Author Contributions

**Hongxiang Yan:** conceptualization, formal analysis, investigation, methodology, visualization, wiring–original draft. **Mark S. Wigmosta:** supervision, funding acquisition, project administration, writing–review and editing. **Ning Sun:** methodology, writing–review and editing. **Song Gao:** investigation, writing–review and editing. **Michael H. Huesemann:** supervision, writing–review and editing.

## Supporting information

Supporting information.

## Data Availability

The data that support the findings of this study are available from the corresponding author upon reasonable request.
