# Supplementary material for: Enhancing Biomass Productivity by Forecast‐Informed Pond Operations
Source: Biotechnol Bioeng. 2025 Feb 7;122(5):1245–57. doi: 10.1002/bit.28952 (PMC11975164; doi:10.1002/bit.28952)
Supplement: Supplementary file 1 — Supporting information. [file BIT-122-1245-s001.docx]

**Supporting Information (SI) Appendix**

**Enhancing Biomass Productivity by Forecast-Informed Pond Operations**

Hongxiang Yan^1*^, Mark S. Wigmosta^1,2^, Ning Sun^1^, Song Gao^3^, and Michael H. Huesemann^3^

^1^ Energy and Environment Directorate, Pacific Northwest National Laboratory, Richland, Washington 99352, United States

^2^ Department of Civil and Environmental Engineering, University of Washington, Seattle, Washington 98195, United States

^3^ Marine Sciences Laboratory, Pacific Northwest National Laboratory, Sequim, Washington 98382, United States

*Corresponding author. E-mail: hongxiang.yan@pnnl.gov

**Table of Contents**

[**Appendix A: Biomass Assessment Tool (BAT)** 3](#_Toc187092098)

[**Appendix B: Huesemann Algae Biomass Growth Model (BGM)** 5](#_Toc187092099)

[**Appendix C: Modular Aquatic Simulation System in Two Dimensions (MASS2)** 7](#_Toc187092100)

[**Appendix D: BGM Validation** 9](#_Toc187092101)

[**Appendix E: Global Ensemble Forecast System (GEFS)** 11](#_Toc187092102)

[**References** 13](#_Toc187092103)

**Appendix A: Biomass Assessment Tool (BAT)**

The BAT is an integrated platform designed for modeling, analysis, and data management to assess national-scale resource and algal biomass production potential in both open pond and closed system facilities. Operating at a high spatial and temporal resolution (e.g., 30–500 m and hourly time step) across the Continental United States (CONUS), the BAT integrates various models and analyses. As detailed in previous studies (Wigmosta et al., 2011; Coleman et al., 2014; Xu et al., 2020), the BAT includes 1) a microalgae growth model: Huesemann Algae Biomass Growth Model (BGM) (Huesemann et al., 2016), 2) a two-dimensional hydrodynamic mass and energy balance model: Modular Aquatic Simulation System in Two Dimensions (MASS2) (Perkins and Richmond, 2004), 3) a multiscale land suitability model, 4) tradeoff analysis tools for evaluating biomass production potential with available land and water resources, 5) water source and use intensity analysis, 6) models for nutrient and CO2 flue gas sources, availability, and demand, 7) least-cost transport models for water, nutrients, CO2, and refinery access, 8) a land valuation and acquisition model, and 9) a site leveling model. Table 1 shows the spatial data sources and descriptions used in the BAT (Wigmosta et al., 2011). The high-resolution spatiotemporal BAT addresses fundamental questions about biomass production, including optimal locations, resource requirements for nutrients, land, and water, achievable biomass and biocrude yields, and strategies to maximize productivity and efficiency. By evaluating trade-offs across various scenarios, the BAT identifies ideal production sites while considering diverse technological and operational pathways. It incorporates factors such as existing land use/land cover, sustainable and alternative water supplies, multi-modal transportation networks, beneficial waste nutrient resources, and refinery infrastructure. Through the integration of detailed spatiotemporal data with biophysical and geospatial models, the BAT delivers multi-scale analyses that connect key Bioenergy Technologies Office (BETO) and industry research efforts to achieve impactful objectives. For more details, we refer to Wigmosta et al. (2011) and Davis et al. (2024).

**Table S1**. Spatial data sources and descriptions used in the BAT.

| **Num.** | **Dataset** | **Description** | **Source** |
| --- | --- | --- | --- |
| 1 | 30m digital elevation model | Elevation data providing the basis for slope and land suitability analysis | U.S. Geological Survey |
| 2 | Hydrography | Surface water delineations including streams, rivers, canals, lakes, and other open water. | U.S. Geological Survey |
| 3 | National land cover database | Standardized 25-category land cover classification | Multi-Resolution Land Characteristics Consortium |
| 4 | Urban area boundaries | Delineation of concentrated populations ≥50,000 | U.S. Census Bureau |
| 5 | Federal or state land ownership | Federal and state protected areas such as national and state parks and monuments, designated wilderness areas, and wildlife refuges | U.S. Geological Survey |
| 6 | National wetlands inventory | Wetland delineation and classification | U.S. Fish and Wildlife Service |
| 7 | Road network | Interstate, major highways, secondary highways, and street level data | Environmental Systems Research Institute |
| 8 | Airport locations | Point locations of all major or minor airports | U.S. Bureau of Transportation Statistics |
| 9 | Environmentally protected/sensitive areas | Compilation of terrestrial and aquatic protected and environmentally sensitive areas | World Database on Protected Areas |
| 10 | Gridded climate | long-term hourly climate data for minimum temperature, maximum temperature, dewpoint, precipitation, etc. | North-American Land Data Assimilation System-2 |

**Appendix B: Huesemann Algae Biomass Growth Model (BGM)**

The BGM calculates the biomass growth rate in algae cultures based on incident light intensity (i.e., PAR) and the culture temperature. Since the strain was parameterized in a sterile, nutrient-replete BG-11 medium at a pH of 7.0, the BGM is specifically suited for pond cultures maintained under similar conditions—nutrient-replete, near-neutral pH, and free from growth inhibitors such as invasive species. In the BGM framework, the outdoor pond volume is divided vertically into multiple layers of equal volume for modeling purposes. In each layer, the biomass concentration $B$ is assumed to increase exponentially in a time interval $\Delta t$ as:

|  | $B\left( t+\Delta t \right)=B\left( t \right)e^{\mu\Delta t}$ | (1) |
| --- | --- | --- |

where $\mu$ is the biomass growth rate in the respective layer. The biomass growth rate $\mu$, determined by both light intensity and water temperature, is calculated as:

|  | $\mu=f(T, I)$ | (2) |
| --- | --- | --- |

where $T$ is the water temperature and $I$ is the light intensity. As each microalgae strain has a unique response to the combination of light and temperature, the response function $f$ is strain‐specific and needs to be experimentally determined before running the model. According to the Beer–Lambert Law (Ingle and Crouch, 1988), for a given biomass concentration, light intensity attenuates as a function of light penetration distance as:

|  | $I\left( z \right)=I_{0}e^{-k_{a}Bz}$ | (3) |
| --- | --- | --- |

where $I\left( z \right)$ is the light intensity at depth $z$, $I_{0}$ is the light intensity at the surface, $k_{a}$ is the biomass light absorption coefficient, and $B$ is the biomass concentration. To account for possible light scattering in dense biomass cultures, the BGM used an experimentally determined scatter-corrected biomass light attenuation coefficient $k_{sca}$ for prediction of light intensity as a function of light penetration distance and biomass concentration (Suh and Lee, 2003):

|  | $k_{sca}=k_{a}\frac{k_{B}}{k_{B}+B}\frac{k_{z}}{k_{z}+z}$ | (4) |
| --- | --- | --- |

where $k_{B}$ and $k_{z}$ are the light scattering coefficients associated with biomass concentration $B$ and light penetration distance $z$. Measurement of $k_{sca}$ is done in a white translucent container mixed from below with a magnetic stirrer and illuminated from above with a multi‐color LED panel simulating sunlight at ca. 2000 µmol/m^2^/s. The outdoor pond will lose biomass at night through dark respiration. The rate of biomass loss $\mu_{dark}$ in the nighttime is estimated as a function of pond water temperature $T$ and the average light intensity $I_{avg}$ during the preceding day as:

|  | $\mu_{dark}=f(T,I_{avg})$ | (5) |
| --- | --- | --- |

The $I_{avg}$ is estimated by averaging the depth‐integrated light attenuation profiles for each time interval $\Delta t$ over the entire day preceding the night. Like the biomass growth rate $\mu$, the biomass loss rate $\mu_{dark}$ is also determined by laboratory experiments. For more details, we refer to Huesemann et al. (2016).

**Appendix C: Modular Aquatic Simulation System in Two Dimensions (MASS2)**

The MASS2 is used to estimate water temperature and evaporative water loss at the pond facility scale. The model uses a structured multi-block, curvilinear computational mesh to represent the channel geometry. Finite volume methods are used to discretize and solve the conservation equations for mass, momentum, and water quality constituents. Water temperature in the ponds is computed by MASS2 using the following depth‐averaged equation that is derived by applying the principle of conservation of energy to a fluid volume as (Patankar, 1980):

|  | $h_{1}h_{2}\frac{\partial\left( dT \right)}{\partial t}+\frac{\partial\left( h_{2}dUT \right)}{\partial\xi}+\frac{\partial\left( h_{1}dVT \right)}{\partial\eta}=\frac{\partial}{\partial\xi}\left( h_{2}\frac{\varepsilon_{1}}{h_{1}}d\frac{\partial T}{\partial\xi} \right)+\frac{\partial}{\partial\eta}\left( h_{1}\frac{\varepsilon_{2}}{h_{2}}d\frac{\partial T}{\partial\eta} \right)+\frac{h_{1}h_{2}H}{\rho c_{v}}$ | (6) |
| --- | --- | --- |

where $h_{1}$ and $h_{1}$ are curvilinear grid metric coefficients in the $\xi$ and $\eta$ directions, $U$ and $V$ are the depth-averaged velocities in the $\xi$ and $\eta$ directions, $d$ is the water depth, $\varepsilon_{1}$ and $\varepsilon_{2}$ are the turbulent eddy diffusion coefficients in the $\xi$ and $\eta$ directions, $H$ is the net surface heat flux at the water surface, $\rho$ is the water density, and $c_{v}$ is the specific heat of the water. The net surface heat flux is estimated as:

|  | $H=H_{s}+H_{a}-(H_{b}+H_{e}+H_{c})$ | (7) |
| --- | --- | --- |

where $H_{s}$ is the net solar shortwave radiation, $H_{a}$ is the net atmospheric longwave radiation, $H_{b}$ is the longwave back radiation, $H_{e}$ is the latent heat flux, and $H_{c}$ is the sensible heat flux. The MASS2 model parameters (e.g., bed depth, conduction rate) were adjusted to represent the elevated outdoor pond conditions (Yan et al., 2023). Evaporative water loss $E$ is estimated as:

|  | $E=\frac{H_{e}}{\rho\lambda_{v}}$ | (8) |
| --- | --- | --- |

where $\lambda_{v}$ is the latent heat of vaporization. Evaporative loss is a highly nonlinear process driven by local meteorological conditions and pond characteristics. These water‐energy interactions are simulated in detail by the open pond temperature model, which provides hourly evaporative loss. For more details, we refer to Perkins and Richmond (2004).

**Appendix D: BGM Validation**

Figure S1 presents the hourly biomass simulations and observations for two climate-simulation ponds under both batch growth and semi-continuous growth modes with two different dilution rates (Huesemann et al., 2016). The BGM is driven by the measured hourly PAR and water temperature, using growth parameters specific to *Chlorella sorokiniana*. The sawtooth pattern observed in the model-predicted concentration curve illustrates the periodic increase of biomass during daylight hours, followed by a decrease at night due to dark respiration. In addition to the simulation curves, the figure includes the NSE (Nash-Sutcliffe efficiency) metric (Nash and Sutcliffe, 1970) values, which indicate the accuracy of the model predictions. The NSE metric is widely used in hydrometeorological research and practice globally. It ranges from ‒∞ to 1, where a value of 1 indicates a perfect fit between observations and simulations, and a value ≤ 0 suggests that the mean of the observed values is a better predictor than the model predictions. The NSE is calculated as follows:

|  | $NSE=1-\frac{\sum_{t=1}^{N} \left( O_{t}-P_{t} \right)^{2}}{\sum_{t=1}^{N} \left( O_{t}-\bar{O} \right)^{2}}$ | (9) |
| --- | --- | --- |

where $N$ is the total number of observations, $O_{t}$ is the observation at time step $t$, $P_{t}$ is the model simulation at time step $t$, and $\bar{O}$ is the mean of the $N$ observations. The BGM shows very high accuracy, with NSE values of 0.99 for both ponds under batch growth mode, and 0.96 and 0.94 for the two ponds under semi-continuous mode with different dilution rates. Based on these fidelity results, the BGM is shown to be highly capable of reproducing the outdoor growth of *Chlorella sorokiniana*.

**
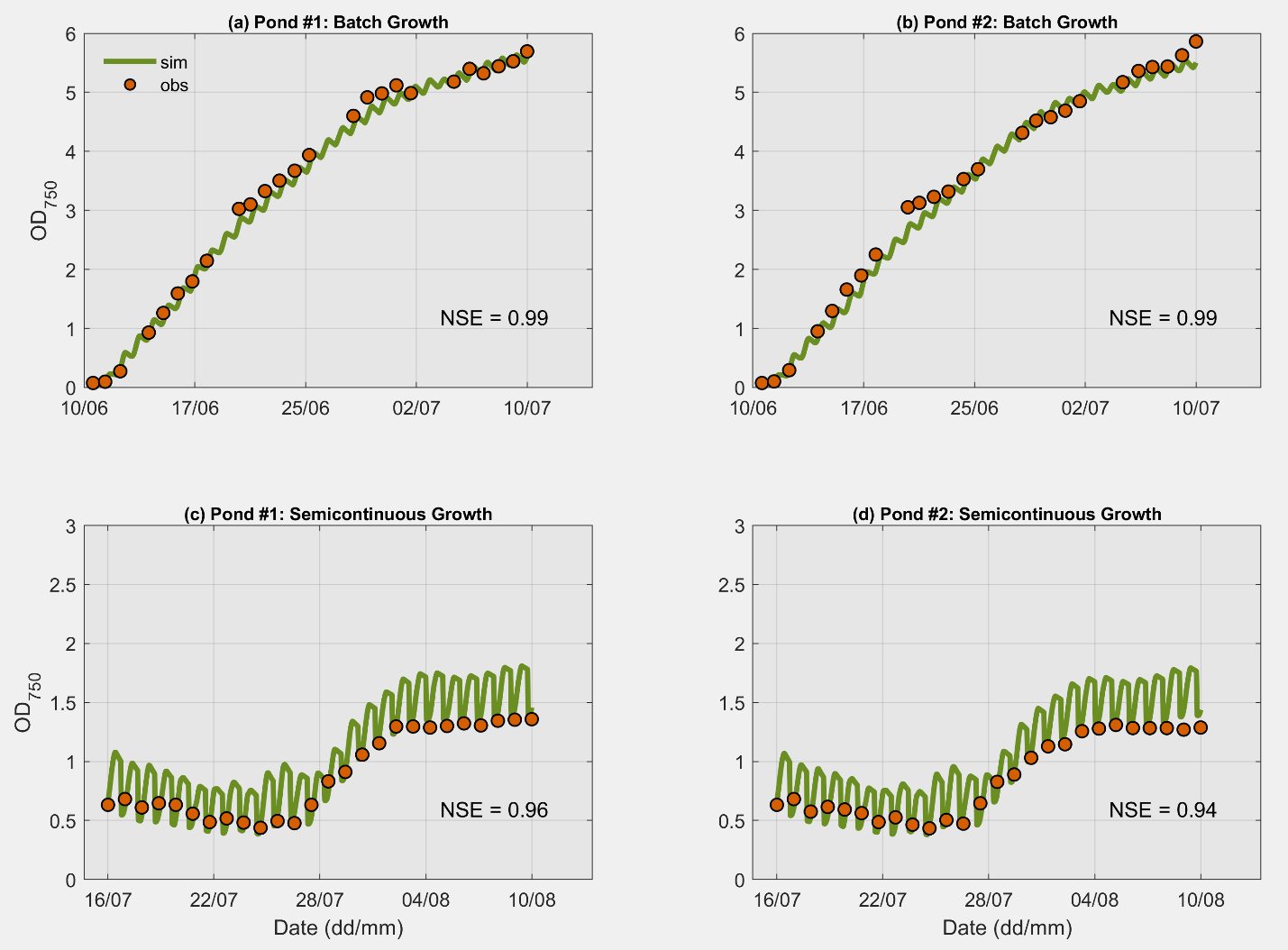
**

Figure S1. The simulated and measured biomass concentrations (OD_750_) in the two climate-simulation ponds. The model was validated across both batch phases (a, b) and two semi-continuous culture phases (c, d).

**Appendix E: Global Ensemble Forecast System (GEFS)**

The GEFS is a numerical weather prediction system developed by the National Centers for Environmental Prediction (NCEP) to produce probabilistic weather forecasts (Hamill et al., 2022). GEFS employs an ensemble approach, running multiple simulations of the same forecast with slightly varied initial conditions and model physics. This method accounts for uncertainties in the atmosphere's initial state and the inherent variability in the modeling process. For this study, we utilized the recently released 2nd-Generation National Oceanic and Atmospheric Administration (NOAA) global ensemble reforecast dataset (Hamill et al., 2013, 2019), which is consistent with the operational 0000 UTC cycle of the currently active GEFS. The dataset consists of an 11-member ensemble, including one control run and ten perturbed members, initialized daily at 0000 UTC. Each reforecast extends up to 16 days, providing middle-range probabilistic forecasts. The GEFS reforecast dataset was developed by NOAA’s Earth System Research Laboratory (ESRL) using version 9.0.1 of the operational GEFS. The reforecasts were initialized with the Climate Forecast System Reanalysis (CFSR) through February 20, 2011, and with the Gridpoint Statistical Interpolation (GSI) analysis system for later periods. This dataset has been available since December 1, 1984, providing a valuable long-term archive for research and operational applications (Li et al., 2016). The initial conditions for the reforecasts were derived from reanalysis data, while perturbations were created using the ensemble transform with rescaling technique. These datasets are instrumental in identifying and addressing systematic errors in forecasts, especially for relatively rare events or long-lead predictions. By leveraging historical consistency in ensemble forecasts, the reforecasts enhance the reliability of predictions across various timescales. The GEFS reforecast data used in this study is publicly accessible at <https://noaa-gefs-retrospective.s3.amazonaws.com/index.html>. For a comprehensive description of the dataset's construction and data availability, refer to Hamill et al. (2019).

**References**

Coleman, A.M., Abodeely, J.M., Skaggs, R.L., Moeglein, W.A., Newby, D.T., Venteris, E.R., Wigmosta, M.S., 2014. An integrated assessment of location-dependent scaling for microalgae biofuel production facilities. Algal Res. 5, 79–94. https://doi.org/10.1016/j.algal.2014.05.008

Davis, R., Hawkins, T., Coleman, A., Gao, S., Klein, B., Wiatrowski, M., Zhu, Y., Xu, Y., Snowden-Swan, L., Valdez, P., Zhang, J., Singh, U., Ou, L., 2024. Economic, Greenhouse Gas, and Resource Assessment for Fuel and Protein Production from Microalgae: 2022 Algae Harmonization Update. Golden, CO (United States). https://doi.org/10.2172/2318964

Hamill, T.M., Bates, G.T., Whitaker, J.S., Murray, D.R., Fiorino, M., Galarneau, T.J., 2019. A Description of the 2nd-Generation NOAA Global Ensemble Reforecast Data Set. NOAA Earth System Research Lab, Physical Sciences Division, Boulder, Colorado, USA.

Hamill, T.M., Bates, G.T., Whitaker, J.S., Murray, D.R., Fiorino, M., Galarneau, T.J., Zhu, Y., Lapenta, W., 2013. NOAA’s Second-Generation Global Medium-Range Ensemble Reforecast Dataset. Bull. Am. Meteorol. Soc. 94, 1553–1565. https://doi.org/10.1175/BAMS-D-12-00014.1

Hamill, T.M., Whitaker, J.S., Shlyaeva, A., Bates, G., Fredrick, S., Pegion, P., Sinsky, E., Zhu, Y., Tallapragada, V., Guan, H., Zhou, X., Woollen, J., 2022. The Reanalysis for the Global Ensemble Forecast System, Version 12. Mon. Weather Rev. 150, 59–79. https://doi.org/10.1175/MWR-D-21-0023.1

Huesemann, M.H., Crowe, B., Waller, P., Chavis, A., Hobbs, S., Edmundson, S., Wigmosta, M., 2016. A validated model to predict microalgae growth in outdoor pond cultures subjected to fluctuating light intensities and water temperatures. Algal Res. 13, 195–206. https://doi.org/10.1016/j.algal.2015.11.008

Ingle, J.D., Crouch, S.R., 1988. Spectrochemical Analysis. Prentice-Hall, Englewood Cliffs, NJ.

Li, W., Wang, Z., Peng, M.S., 2016. Evaluating Tropical Cyclone Forecasts from the NCEP Global Ensemble Forecasting System (GEFS) Reforecast Version 2. Weather Forecast. 31, 895–916. https://doi.org/10.1175/WAF-D-15-0176.1

Nash, J.E., Sutcliffe, J.V., 1970. River flow forecasting through conceptual models part I — A discussion of principles. J. Hydrol. 10, 282–290. https://doi.org/10.1016/0022-1694(70)90255-6

Patankar, S. V., 1980. Numerical Heat Transfer and Fluid Flow. Hemisphere, New York, NY.

Perkins, W.A., Richmond, M.C., 2004. MASS2, Modular Aquatic Simulation System in Two Dimensions: Theory and Numerical Methods. Rep. PNNL-14820-1, Pacific Northwest National Laboratory, Richland, Washington.

Suh, I.S., Lee, S.B., 2003. A light distribution model for an internally radiating photobioreactor. Biotechnol. Bioeng. 82, 180–189. https://doi.org/10.1002/bit.10558

Wigmosta, M.S., Coleman, A.M., Skaggs, R.J., Huesemann, M.H., Lane, L.J., 2011. National microalgae biofuel production potential and resource demand. Water Resour. Res. 47, W00H04. https://doi.org/10.1029/2010WR009966

Xu, H., Lee, U., Coleman, A.M., Wigmosta, M.S., Sun, N., Hawkins, T., Wang, M., 2020. Balancing Water Sustainability and Productivity Objectives in Microalgae Cultivation: Siting Open Ponds by Considering Seasonal Water-Stress Impact Using AWARE-US. Environ. Sci. Technol. 54, 2091–2102. https://doi.org/10.1021/acs.est.9b05347

Yan, H., Wigmosta, M.S., Huesemann, M.H., Sun, N., Gao, S., 2023. An ensemble data assimilation modeling system for operational outdoor microalgae growth forecasting. Biotechnol. Bioeng. 120, 426–443. https://doi.org/10.1002/bit.28272
